# Histopathology of the Tongue in a Hamster Model of COVID-19

**DOI:** 10.21203/rs.3.rs-4590482/v1

**Published:** 2024-07-04

**Authors:** John M Coggins, Marina Hosotani Saito, Rebecca Cook, Shinji Urata, Megumi Urata, Nantian Lin Harsell, Wilhelmina Nanrui Tan, Bibiana Toro Figueira, Megan Bradley, Nadia Z. Quadri, Janisah Amirah I. Saripada, Rachel A. Reyna, Junki Maruyama, Slobodan Paessler, Tomoko Makishima

**Affiliations:** University of Texas Medical Branch; University of Texas Medical Branch; University of Texas Medical Branch; University of Texas Medical Branch; University of Texas Medical Branch; University of Texas Medical Branch; University of Texas Medical Branch; University of Texas Medical Branch; University of Texas Medical Branch; University of Texas Medical Branch; University of Texas Medical Branch; University of Texas Medical Branch; University of Texas Medical Branch; University of Texas Medical Branch; University of Texas Medical Branch

**Keywords:** SARS-CoV-2, dysgeusia, hamster

## Abstract

**Objective:**

With altered sense of taste being a common symptom of coronavirus disease 2019 (COVID-19), our objective was to investigate the presence and distribution of severe acute respiratory syndrome coronavirus-2 (SARS-CoV-2) within the tongue over the course of infection.

**Methods:**

Golden Syrian hamsters were inoculated intranasally with SARS-CoV-2 and tongues were collected at 2, 3, 5, 8, 17, 21, 35, and 42 days post-infection (dpi) for analysis. In order to test for gross changes in the tongue, the papillae of the tongue were counted. Paraffin-embedded thin sections of the tongues were labeled for the presence of SARS-CoV-2 antigen.

**Results:**

There was no difference in fungiform or filiform papillae density throughout the course of infection. SARS-CoV-2 antigen was observed in the circumvallate papillae taste buds (3–35 dpi) and autonomic ganglia (5–35 dpi), as well as in the serous and mucous salivary glands of the posterior tongue (2–42 dpi).

**Conclusion:**

The presence and distribution of SARS-CoV-2 suggest that the virus could cause taste disturbance by infecting the circumvallate taste buds. This effect could be exacerbated by a diminished secretion of saliva caused by infection of the serous salivary glands and the autonomic ganglia which innervate them.

## Background

Severe acute respiratory syndrome coronavirus-2 (SARS-CoV-2) infection can cause anosmia and ageusia with sizeable prevalence rates of 38.2% and 36.6%, respectively.^1,2^ These symptoms have played significant roles in the epidemiology of the COVID-19 pandemic as they are early manifestations of the disease and allow the infected to be identified before traditional viral respiratory symptoms are noted.^3–5^ In some cases, they may be the sole manifestations of COVID-19, further enhancing our ability to contact trace and quickly quarantine those infected.^6,7^ In light of this, the Centers for Disease Control and Prevention (CDC) added loss of taste and smell to the list of COVID-19 symptoms in April of 2020. While anosmia and ageusia serve significant epidemiological purposes, they are equally significant in their affront on quality of life. The loss of taste and smell can be distressing and is associated with increased rates of depression and suicidal ideation.^8^ The median duration for COVID-19 related anosmia and dysgeusia is seven days; however, some individuals report these symptoms for significantly greater periods of time.^9^ Moreover, vaccination has been shown to not prevent or mitigate the onset of anosmia.^10^

**Figure 1:**
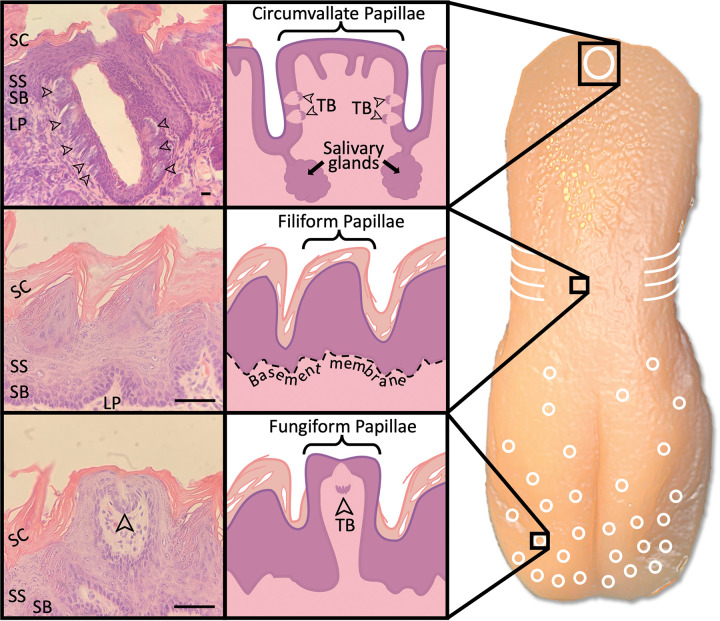
Anatomy of the Hamster Tongue. Distribution of tongue papillae and related histology. A midline circumvallate (CV) papilla resides in the posterior region (white oval), foliate on the sides (white curved lines), fungiform on the anterior (white circles), and filiform filling the remaining space. Taste buds (arrowheads) reside on the sides of the CV papillae and the tips of the fungiform papillae but are not found on the filiform papillae. Salivary glands are located beneath the troughs of CV papillae and help dissolve tastants. SC;stratum cornuem, SS; stratum spinosum, SB; stratum basale, LP; lamina propria, TB; taste buds

The sensation of taste is dependent upon multiple structures and processes. To begin, salivary glands produce saliva to dissolve tastants.^11^ The parotid, submandibular, and sublingual salivary glands produce the majority of saliva but are supplemented by the serous salivary glands of von Ebner, which are located directly underneath the furrows of the circumvallate (CV) papillae (Fig. 1).^11^ Once dissolved, tastants enter taste buds through taste pores and bind to their respective taste cell receptors.^11^ A signal cascade converts the chemical signal to a neural impulse, which then travels to the brain.^11^ These signals are heavily supplemented by the sense of smell, which is often purported to contribute 75–95% of the perceived sense of taste; however, given the difficulty in quantifying perceived taste sensation, these numbers have undergone little empirical validation.^12^ The loss of function of any of these structures is capable of causing taste disturbance.^11^ In fact, ageusia boasts a wide variety of etiologies, including drug-induced, age-related, neuropathic, dietary deficiency, systemic condition, and iatrogenic (radiation, chemotherapy, or surgery), and the pathophysiology for each varies according to its etiology.^13^ For example, the loss of saliva in Sjögren’s syndrome disrupts the dissolution of tastants and decreases taste sensation, while zinc deficiency causes ageusia by limiting the production of important salivary enzymes.^13,14^

SARS-CoV-2 may cause ageusia through a variety of processes. One hypothesis postulates that SARS-CoV-2 induces ageusia by activating toll-like receptors and interferon receptors in the taste buds, stimulating inflammatory processes and impeding taste cell regeneration.^15^ A separate hypothesis suggests that SARS-CoV-2 could have an effect on the nervous system.^16^ Studies have shown that SARS-CoV-2 is capable of using the trigeminal and olfactory nerves to travel into the central nervous system. Therefore, COVID-19 may cause ageusia by eliciting T cell-mediated autoimmune damage and demyelination while traveling through nerves.^16^ Lastly, the angiotensin converting enzyme 2 (ACE2) receptor has been implicated in the pathophysiology of taste dysfunction.^17^ Taste dysfunction is a rare side effect of ACE inhibitor therapy, and SARS-CoV-2 downregulation of ACE2 may cause dysgeusia through a similar mechanism.^18,19^ A better understanding of which structures are affected by the SARS-CoV-2 virus could help explain the pathophysiology of SARS-CoV-2 related taste dysfunction. This study aims to reduce this gap in knowledge by determining the location of the SARS-CoV-2 virus within the tongue over the course of an infection.

## Materials and Methods

### Animal Experiment:

We chose a hamster model for our study because golden Syrian hamsters are susceptible to direct clinical isolates of SARS-CoV-2 virus without the need of genetic modifications,^20–22^ the hamsters are immunocompetent and feature low mortality with significant lung pathology, and they are previously well characterized for olfactory studies.^23,24^ Additionally, hamster models have been historically utilized to study taste physiology.^25^ The hamster tongues used in this study were derived from a study on anosmia after SARS-CoV-2 infection; details are published in a manuscript by Reyna et al (2022).^24^ Briefly, five to six-week-old female Golden Syrian hamsters were inoculated intranasally with 100 μL of 10^5^ median tissue culture infectious dose (TCID_50_) of SARS-CoV-2 alpha-strain diluted with phosphate-buffered saline or a vehicle control. The infected hamsters were divided into 8 groups (n = 4 each) with tongues harvested at 2, 3, 5, 8, 17, 21, 35, and 42 days post-infection (dpi). In this study chemosensory deficit in olfaction was confirmed in infected hamsters by measuring the duration of time required to find a buried cookie. The hamsters showed significant anosmia at days 2, 3, and 5 dpi (days post-infection) that progressively recovered through 42 dpi as shown by the food detection test.^24^ All animal studies were reviewed and approved by the Institutional Animal Care and Use Committee at UTMB and were conducted according to the National Institutes of Health guidelines.

### Tissue Processing:

The whole tongue was dissected and fixed in 10% formalin for more than 7 days. The tongue was cut in the center sagittally and each half embedded in paraffin. Tissue was dehydrated in ethanol, cleared in xylene, and embedded in paraffin in the standard fashion. Tongues were then sectioned with sagittal cuts at a thickness of 5 μm. Slides were stained with hematoxylin and eosin (H&E) or labeled with SARS Nucleocapsid protein antibody (Novus Cat# NB100–56576, RRID:AB_838838).

### Papillae Counting:

**Figure 2:**
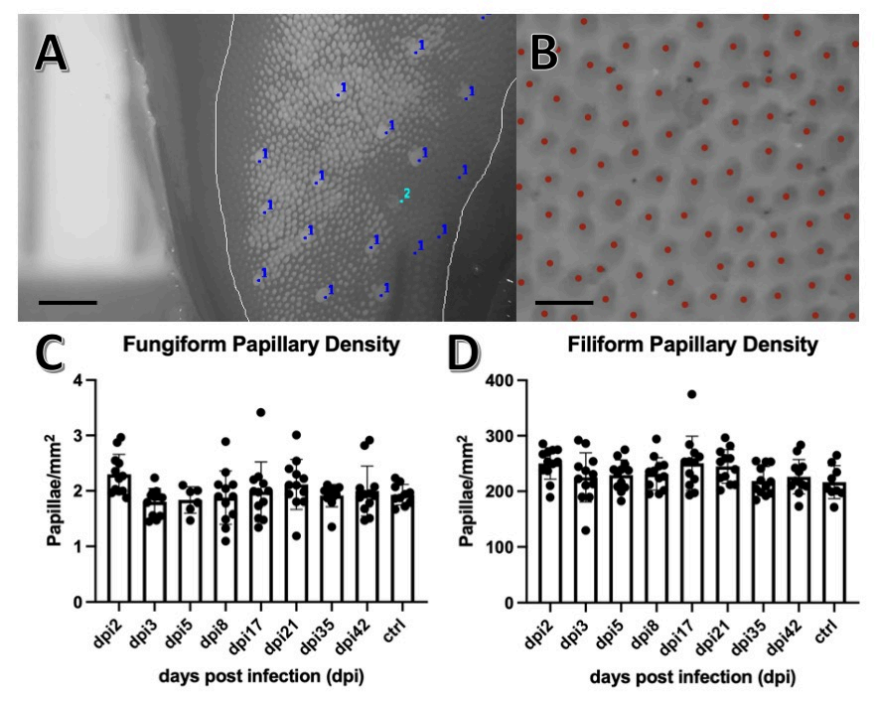
Papillae Counting. **A)** Magnified 8-bit image of the anterior tongue using Image J v1.53a. The region of interest (ROI) was demarcated (grey line) using Image J freehand tool, the fungiform papillae were counted using the Cell Counter plugin for Image J (dark blue dots), and the area (mm^2^) calculated using the measurement tool in Image J with the ruler behind the tongue for reference. Papillae were excluded from the count (light blue dot) if they were found to not be a complete circle, not completely within the ROI, or not approximately 3:1 in size when compared with surrounding filiform papillae. Scale bar indicates 500μm. **B)** Magnified 8-bit inverted image of the anterior tongue using Image J v1.53a. Red dots indicate computer generated counting of the papillae using the Image-based Tool for Counting Nuclei (ITCN) plugin for Image J. Scale bar indicates 100μm. **C)** Fungiform papillae densities were plotted against days post infection (dpi). Error bars indicate mean and standard deviation. No significant differences were found between any of the infected groups (dpi#) or control (ctrl) when tested with ANOVA set to p < 0.05. For all groups, n = triplicates of 4. **D)** Filiform papillae densities were plotted against days post infection (dpi). Error bars indicate mean and standard deviation. No significant differences were found between any of the infected groups (dpi#) or control (ctrl) when tested with ANOVA set to p < 0.05. For all groups, n = triplicates of 4.

Formalin fixed hamster tongues were dyed with green food coloring (Betty Crocker Classic Gel Food Color) diluted in phosphate buffered saline to facilitate visualization of the papillae. Images of the anterior, middle, and posterior sections of each tongue were captured using a dissecting microscope at 25x magnification (Fig. 2). Images were converted to 8-bit black and white. Using a ruler placed in the background of the image, the scale of the image in millimeters was determined using the set scale function in Image J v1.53a. The portion of the tongue within focus was demarcated as the region of interest (ROI) using the freehand tool, and the area was measured using the measure function in Image J. Fungiform papillae within the ROI were then individually counted using the Cell Counter plugin for Image J v1.53a. Fungiform papillae were counted if they met the following criteria: the papillae were complete circles, larger than filiform papillae in an approximate ratio of 3:1, and located completely within the selected ROI. Fungiform papillae were counted within three distinct ROI using the image of the anterior tongue to create a triplicate measurement. The number of papillae and area within each section was added, and the density was calculated. Filiform papillae were counted using one ROI within each of the three sections of the tongue (anterior, middle, and posterior) using the automated counter Image-based Tool for Counting Nuclei (ITCN) plugin for Image J. The estimated width of each papilla was set to 30 pixels, and the minimum distance between papillae was set to 15 pixels. The automated counter was set to detect light peaks within the selected ROI. This process was repeated twice more to produce triplicate measurements for each tongue.

### Immunohistochemistry:

Thin-sectioned tongue tissue was deparaffinized, rehydrated, and subjected to heat-induced antigen retrieval in Target Retrieval Solution (S-1700, Dako). The slides were incubated with SARS Nucleocapsid Protein antibody (1:200 dilution for Supp. Figure 1 and Supp. Table 1, 1:1,000 dilution for Supp. Figure 2 and Supp. Table 2) (Novus Biologicals Cat# NB100–56576, RRID:AB_838838) followed by incubation with a biotinylated secondary antibody (SignalStain Boost IHC Detection Reagent HRP Rabbit, Cell Signaling Technology). Lastly, slides were developed with 3,3’-Diaminobenzidine (DAB) (ImmPACT DAB Substrate, Peroxidase HRP, Vector Laboratories) and counterstained with hematoxylin. In addition, four slides were stained without primary antibody in order to test non-specific binding of the secondary antibody. All four slides from dpi 5, dpi 17, and mock which included all structures of interest did not show any non-specific false positive staining in the tongue.

### Histopathology grading:

H&E stained and SARS-CoV-2 antibody labeled slides were graded by at least three independent observers blinded to the specimen information. Nine structures within the tongue were evaluated: the autonomic ganglia, serous salivary glands, mucous salivary glands, fungiform papillae, filiform papillae, circumvallate (CV) papillae, muscle, nerves, and vasculature. Graders evaluated H&E slides for de-epithelialization, taste bud disengagement, fibroblast cell infiltrate, and blood cell infiltrate within each of the eight structures (Supp. Figure 3). The number of cells within the single largest CV taste bud, fungiform taste bud, and autonomic ganglia was recorded. Cells with visible nuclei within the structure were counted. Structures labeled with SARS-CoV-2 antibody were graded for the intensity of cell labeling on a scale of 0–2, with 0 indicating no staining, 1 mild staining, and 2 strong staining (Supp. Figure 4) (Supp. Table 1 and Supp. Table 2). If a structure was not identified within a tongue, the structure was not graded and did not contribute to the overall score (Supp. Table 3).

### Statistics:

Papillary densities were compared among different timepoints and animals with a one-way ANOVA and post hoc Tukey test in Prism v9 with p < 0.05 determining a statistically significant difference.

Scores for SARS-CoV-2 antibody labeling (Supp. Table 1 and Supp. Table 2) from each timepoint (dpi) and mock infected group were compared within each structure of fungiform papillae taste buds, CV papillae taste buds, serous salivary glands, mucous salivary glands, and autonomic ganglia. We had four reviewers in Experiment 1 (1:200 dilution antibody)(Supp. Table 1) and three reviewers in Experiment 2 (1:1000 dilution antibody)(Supp. Table 2). Each reviewer provided a grading score ranging from 0 to 2. We then compared the means of the reviewer scores for each of the timepoints and mock group. Since the data did not follow normality assumption and the sample size was small, we placed non-parametric Kruskal-Wallis test for median score comparison (p < 0.05, significant difference). And for pairwise comparison between each of the timepoints we placed Dwass, Steel, Critchlow-Fligner (DSCF) multiple comparison analysis. The analyses were performed with SAS version 9.4 (SAS, Inc., Cary, NC).

## Results

Counting of the fungiform and filiform papillae indicated there was no change in the number of papillae throughout the course of SARS-CoV-2 infection (Fig. 2). H&E staining revealed no de-epithelialization, taste bud disengagement, or cellular infiltrate at any time point from 2 through 42 dpi. The numbers of cells within the infected CV taste buds (6.4 ± 2.9), fungiform taste buds (7.9 ± 1.8), and autonomic ganglia (6.3 ± 1.7), were not statistically different from the number of cells within the same structures of mock infected control tongues (CV 8.0 ± 1, fungiform 6.8 ± 0.7, autonomic ganglia 7.8 ± 1.2) (mean ± standard deviation).

**Figure 3:**
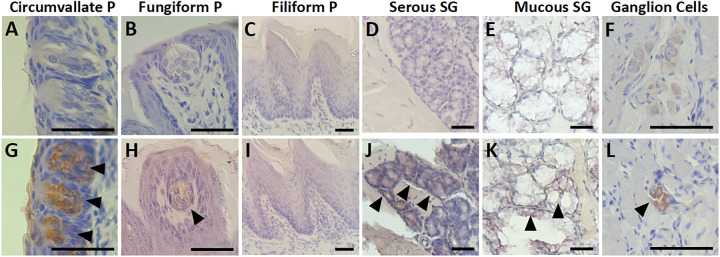
SARS-CoV-2 antigen labeling in the tongue. Representative images of immunohistochemical labeling (1:200 antibody dilution) of structures of interest with or without infection with SARS-CoV-2. The infected samples at 5 dpi are shown in the lower panels (G, H, I, J, K, L), with arrowheads pointing to positive brown-colored labeling of the SARS-CoV-2 antigen, compared with negative labeling in the mock infected control samples in the upper panels (A, B, C, D, E). The circumvallate papilla taste buds (G) and ganglion cells (L) show positive labeling, while fungiform papilla taste bud (H), serous salivary gland cells (J), mucous salivary gland cells (K) show weakly positive labeling. The filiform papilla cells (I) were negative for labeling. P, papillae. SG, salivary gland. Scale bars indicate 100 μm.

Immunohistochemical (IHC) analysis of the hamster tongues revealed the presence of SARS-CoV-2 antigen post-infection in the cytoplasm of cells in the circumvallate papillae, autonomic ganglia, and salivary glands (Fig. 3, Supp. Figure 1, Supp. Figure 2). The autonomic ganglia were labeled positively for the SARS-CoV-2 antigen at 5 dpi through 21 dpi. At 42 dpi, the labeling decreased in the intensity and proportion of cells labeled. The CV papillae taste buds were labeled for SARS-CoV-2 antigen at 2 through 42 dpi, and no strong positive labeling was found in the control hamsters. Serous and mucous salivary glands labeled positive at 2 dpi to 42 dpi. The fungiform papillae taste buds showed weak labeling, most notably at 5 dpi; however, this labeling was considerably less than the labeling in the CV papillae taste buds (Fig. 3G, H). However, SARS-CoV-2 antibody labeling scores did not show any significant difference at any timepoint after infection or when compared with mock control in any of the anatomical structures (p > 0.05) (Supp. Table 1 and Supp. Table 2). SARS-CoV-2 antigen was not detected in the filiform papillae (Fig. 3I), muscle, or vasculature within any samples of the infected or control groups.

## Discussion

Fungiform papillae density is implicated as a proxy for taste sensitivity, with higher densities indicating greater taste sensation,^26^ although some research has brought this into question.^27,28^ Fungiform papillae density is not often used as a metric for acute injury or infection as the offense is unlikely to alter the number of papillae. For this reason, a change in papillae density was not expected. Indeed, papillae densities in the hamster tongues of our study were similar to that at baseline with comparable numbers of papillae across infected and control groups. This experiment provides valuable documentation that the number of fungiform papillae did not decrease as a result of infection. This is important as fungiform papillae can degenerate into filiform papillae when fully denervated, changing the number of papillae.^29^ Moreover, SARS-CoV-2 infection of the tongue can cause damage to the neurite fibers at the base of taste buds potentially influencing the innervation.^30^

Immunohistochemistry analysis revealed the presence of SARS-CoV-2 antigen in three structures: the CV papillae taste buds, autonomic ganglia, and the salivary glands. A viral infection in any of these structures could affect the sense of taste by directly disrupting cell function in the taste buds or by decreasing saliva production.^31,32^ Interestingly, these findings of preferential localization of viral antigen mirror a similar study of the rabies virus in the tongues of dogs, which also found signs of rabies infection in the CV papillae, autonomic ganglia, and serous salivary glands.^33^ The CV papillae house approximately half of all taste buds in the human tongue.^34^ These taste buds reside on the sides of the CV papillae and play a major role in taste sensation. In our experiments, SARS-CoV-2 antigen was consistently and markedly detected in the CV taste buds, indicating that SARS-CoV-2 infects these cells. Autonomic ganglia are found adjacent to nerves within the tongue and control the secretion of saliva from glands within the tongue.^35^ An infection of the autonomic ganglia could reduce saliva production and prevent tastants from dissolving and binding to taste cell receptors. This effect could be augmented by direct infection of the salivary glands. Indeed, the SARS-CoV-2 antigen was detected in the salivary glands throughout our study from 2 through 42 dpi. Decreased saliva contributing as a mechanism of taste loss would be consistent with the COVID-19 clinical picture, as xerostomia is a recognized symptom of the infection.^36^

The primary means of entry into the cell for SARS-CoV-2 is the ACE2 receptor,^37^ which is expressed in the filiform papillae and type 2 taste cells.^38–40^ In our study, SARS-CoV-2 antigen was not detected in the filiform papillae. This result could be related to the thick layer of keratin the filiform papillae maintain, shielding them from viral attack. It is important to note that the filiform papillae, lacking taste buds, do not contribute to the sense of taste, and an infection of these structures would not affect the ability to taste. Type 2 taste cells contain G protein-coupled receptors (GPCRs) for either bitter, umami, or sweet tastants.^41^ These cells are found primarily within the taste buds of the fungiform and CV papillae in humans, whose combined taste buds account for the majority of taste sensation.^11^ In hamsters, circumvallate papillae contribute a smaller percentage (23%) of taste buds to the sense of taste than in humans, with the remaining taste buds being more evenly distributed among the foliate papillae (32%), fungiform papillae (18%), palate (12%), and epiglottis (10%).^42^ Due to the orientation of our sectioning we did not evaluate the foliate papillae histology. In our study, the fungiform papillae taste buds had minimal SARS-CoV-2 antigen labeling throughout the time course. In comparison, the CV papillae taste buds showed marked labeling from 3 through 35 dpi. The infection of these taste buds likely coincide with the ACE2 receptor within the type 2 taste cells leading to a significant effect on the sense of taste.^39^ The infection of CV papillae taste buds by SARS-CoV-2 has been described in studies of the human tongue providing strong evidence for a mechanism of taste disturbance as a direct infection of type 2 taste cells via the ACE2 receptor causing taste bud damage, regional inflammation, and neurite damage.^30,43,44^ In human patients, the combination of CD8 + T-cell responses and neurite damage caused by SARS-CoV-2 infections of type 2 taste cells was associated with taste disturbance.^30^ Patients who continued to have taste disturbance continued to show evidence of viral particles within the type 2 taste cells with regional inflammation and neurite damage.^30^

We speculate that the length of infection in the tongue is crucial for the prolonged taste disturbance. In our hamster model, SARS-CoV-2 was absent from the lungs after 8 dpi, indicating a clearing of the virus within 1 week.^24^ On the other hand, SARS-CoV-2 virus existed within the tongue through 35 dpi, a significantly longer time period, suggesting the possibility of long-lasting viral production within the tongue. In fact, in humans, long-lasting SARS-CoV-2 viral products were identified within human tongues up to 63 days after infection, with duration of viral products within the tongue correlated to the duration of taste disturbance.^30^ Typically, patients develop chemosensory changes 4–5 days after the onset of other symptoms and experience return of taste and smell 7–14 days later.^43^ This time course would be consistent with our findings, as minimal SARS-CoV-2 antigen labeling was noted at 2 dpi which quickly increases thereafter. We did detect persistent positive labeling through 35 dpi in our hamsters, which is beyond the estimated recovery of loss of taste. This discrepancy could occur due to a regain of taste bud function despite a continued presence of viral antigens. Atypically, patients experience prolonged or permanent loss of taste. It is possible that a separate mechanism produces this prolonged version of COVID-19 related taste dysfunction. Conversely, our study’s sample size may have been too small to capture the particular type of infection leading to prolonged taste disturbance.

Our attempt to identify the peak timepoint of infection failed, as SARS-CoV-2 antibody labeling scores showed no statistically significant differences among any of the experimental groups. In both Experiment 1(SARS-CoV-2 antibody 1:200 dilution) (Supp. Table 1) and in Experiment 2 (SARS-CoV-2 antibody 1:1000 dilution) (Supp. Table 2), there was no statistically significant difference in the scores along all timepoints when tested with non-parametric Kruskal-Wallis test (p > 0.05). However, due to the overall small sample size and loss of datapoints (Supp. Table 3), quantification of our dataset and the results should be interpreted with caution. The statistical significance may also be affected by the incomplete penetrance of SARS-CoV-2 related taste disturbance, in which case infected hamsters may not display positive antibody staining within their tongues.

Our COVID-19 hamster model showed deterioration of olfactory function mainly in the early days starting from 2 dpi to at least 5 dpi after infection with SARS-CoV-2 virus alpha strain.^24^ In comparison, we observed labeling with the SARS-CoV-2 nucleocapsid antibody in the tongue in a delayed timeline with CV papillae taste buds starting from 3 dpi and the autonomic ganglia showing the strongest labeling at 5–17 dpi. We speculate this difference is due to the accessibility of the virus to the target cells. The surface of the tongue is covered with thick keratin, which may protect the virus from penetrating into deeper cell layers initially after infection. However, over time, the virus particles that may have migrated through the openings of the salivary glands may be able to spread into deeper structures. Thus, this taste disturbance being a milder manifestation, may not be recognized by the infected humans because of the dominating anosmia.

Our hamster study showed consistent infection of the autonomic ganglia and minor salivary glands within the tongue. The autonomic ganglia of the tongue are peripheral neuronal cell bodies which provide innervation to the minor salivary glands of the tongue. Neuronal cell bodies express ACE2 significantly more than axons and dendrites.^45^ This is consistent with the SARS-CoV-2 antigen labeling within the autonomic ganglia but not within the nerves of the tongue in our hamster model. We speculate that the infection of these structures involved in the production of saliva could constitute a second mechanism for taste disturbance caused by SARS-CoV-2 and that the difference between the short-lived and the prolonged taste disturbances may be due to different mechanisms. The short-lived taste disturbance may be caused by direct changes to the cells with a faster turnover, such as the taste cells and the salivary gland cells, interfering with the delivery of the tastants. Conversely, the prolonged taste disturbance may be determined by the extent of viral penetration into cells with slower or no turnover, such as the autonomic ganglia.

Our study indicated that the pathophysiology of the taste dysfunction caused by SARS-CoV-2 is likely related to the direct infection of the taste cells coupled with decreased saliva production. In light of this, therapies which prevent taste cell damage, maximize taste cell function and regeneration, and treat xerostomia would be most likely to result in improved taste sensation. Few studies have been conducted to trial possible therapies for COVID-19 related taste dysfunction. Of these, a non-randomized control trial identified beneficial effects of oral triamcinolone topical steroid paste,^46^ and cerebrolycin, a mixture of brain peptides previously shown to have neurotrophic and neuroprotective effects.^47^ The mechanism by which SARS-CoV-2 may damage taste cells is unknown, but one possible pathway includes inflammatory cytokines and toll-like receptors.^14^ By this mechanism, steroid therapy may be capable of preventing taste dysfunction, as in the case of triamcinolone paste.^46^ Theophylline is another therapeutic candidate which has been shown to increase taste cell function by augmenting the signaling of GPCRs within taste cells.^48^ Secretagogues like cevimeline hydrochloride hydrate and pilocarpine hydrochloride could treat xerostomia.^49^ Lastly, SARS-CoV-2 is associated with hypozincemia which has been linked to taste dysfunction and xerostomia; therefore supplementation of zinc may be beneficial for maximizing taste cell function and treatment of xerostomia.^50^ Further research is needed to elucidate the pathophysiology of SARS-CoV-2 infection-induced taste dysfunction and guide development of treatment regimens.

## Conclusion

SARS-CoV-2 antigen was detected in the CV papillae taste buds, salivary glands, and autonomic ganglia of the tongue of the hamster model of COVID-19. Our results suggest that the loss of function of the CV papillae taste buds, salivary glands, or autonomic ganglia may individually or in combination result in COVID-19-related taste disturbance.

## Data Availability

All data generated and analysed in this study are included in this published article and its supplementary figures and tables.

## Abbreviations

COVID-19: Coronavirus disease 2019
SARS-CoV-2: SARS-CoV-2Severe acute respiratory syndrome coronavirus-2
Dpi: days post-infection
CDC: Centers for Disease Control and Prevention
CV: circumvallate
ACE2: angiotensin converting enzyme 2
TCID: tissue culture infectious dose
H&E: hematoxylin and eosin
ROI: region of interest
ITCN: Image-based Tool for Counting Nuclei
DAB: Diaminobenzidine
DSCF: Dwass, Steel, Critchlow-Fligner
